# Creativity and resilience: a mini-review on post-pandemic resources for adolescents and young adults

**DOI:** 10.3389/fpubh.2023.1117539

**Published:** 2023-05-24

**Authors:** Aurelia De Lorenzo, Lynda S. Lattke, Emanuela Rabaglietti

**Affiliations:** SE-CREA Research Group, Department of Psychology, University of Turin, Turin, Italy

**Keywords:** creativity, resilience, COVID-19, adolescents, young adults

## Abstract

**Background:**

Two years after the outbreak of the pandemic, several studies look at the consequences for the well-being and mental health of young people. In particular, creativity and resilience are cited in the scientific literature as resources that promote this well-being in adolescents and young adults.

**Purpose:**

This mini-literature review was created with the aim of examining how many articles have explored the relationship between creativity and resilience in adolescents and young adults since the onset of the pandemic.

**Methods:**

Particular attention was paid to how many of the articles actually related to the consequences of the pandemic, in which country they were published, their target population, and the models, instruments and variables used to analyze them.

**Results:**

Only 4 articles emerged from the screening, of which only one was actually related to pandemic consequences. All articles were published in Asian countries with a target group of university students. Three of the articles used mediation models to examine the relationship between resilience as an independent variable and creativity as a dependent variable. All articles used self-assessment instruments for creativity and resilience, both at the individual and group level.

**Significance:**

This mini-review offers us the opportunity to reflect on the lack of studies that have addressed the issue of youth resources in the form of creativity and resilience since the beginning of the pandemic. The results show us a still underdeveloped interest in creativity in the scientific literature, in contrast to what the media reports on the promotion of creativity in daily life.

## 1. Introduction

The pandemic COVID-19 represented a historical change caused by unforeseen events that people had to cope with, although they did not have sufficient psychological resources several studies (1–4) have shown that the consequences of the pandemic had a negative impact on well-being and mental health, especially among adolescents and young adults. Against this background, everyday creativity [also referred to as Mini-C in (5)] emerged as a protective factor that could be strengthened (6–8).

Creativity, defined as the human capacity to generate original, flexible and effective ideas, insights and solutions (9), appears to be an indispensable resource for managing change, generating invention and innovation, and meeting the challenges of our increasingly complex society (5).

Numerous studies have shown that creativity facilitates individual adjustment and growth (10–12), and in the case of trauma or a stressful situation, creativity supports post-traumatic growth (12–14).

Several studies (15–17) also argue that creative thinking contributes to an individual’s well-being by promoting self-actualization (18), self-confidence (15, 19) and greater perceived control over problems in life (20). The COVID-19 pandemic and its consequences for daily life, schooling and work lead us to reconsider creativity within a unique historical period (21). The few studies that have looked at creativity in the early phase of the pandemic suggest that the period of isolation fostered everyday creativity (22, 23) and that creative skills helped people become more resilient and therefore better able to cope and improve their well-being (7, 24).

Resilience is a dynamic developmental process that promotes positive adaptation to stressful, adverse and traumatic circumstances (25). Through resilience, individuals are able to attribute positive meaning to complex events, cope with negative emotions and adapt to external stressors that change throughout life (26). Creativity is one of the factors that promote resilience (27, 28). Resilient and creative people share personal characteristics such as flexibility, resourcefulness, adaptability and originality (13), as well as a number of contextual factors such as family support and community (29). Literature in the last 2 years has highlighted the role of creativity as a potential protective factor for individual and group resilience during prolonged periods of isolation (30). In addition, providing people with tools to be more creative has been shown to strengthen resilience and increase the number of resilient behaviors and daily creative activities (27). The interaction between creativity and resilience therefore promotes the emergence of protective factors that support people to achieve positive outcomes even in adverse situations (5, 31).

Two years after the spread of COVID-19, young people are the most affected population group (32–34). Studies from the last 2 years show that adolescents and young adults are experiencing worrying levels of depression, anxiety and lower life satisfaction, with mental health problems increasing significantly in adolescents compared to pre-pandemic levels (35–37).

Adolescence and young adulthood are characterized by intense biological, cognitive and psychosocial changes that affect the reorganization of identity and the development of creativity (38). At this stage of development, such identity reordering can be seen as a creative process in itself, with adolescents and young adults exploring multiple alternatives (38). Creativity can be seen as a kind of resilience mechanism to cope with the anxiety and stress associated with adolescents’ and young adults’ developmental tasks (38). Therefore, promoting creative expression can be an effective way to frame these stressors in a more adaptive way (39). Based on these theoretical premises, this literature review aims to examine the quantity and type of articles published since the onset of the pandemic (2020) to describe and explore the relationships between creativity and resilience in adolescents and young adults. Specific objectives of the study are to:

1. Assess how many articles have addressed creativity and resilience in relation to the post-pandemic situation
2. Determine the geographic origin (country) of the publications dealing with creativity and resilience
3. Assess how many articles have measured creativity and resilience with a target group of adolescents or young adults
4. Describe how the constructs of creativity and resilience and any other related variables have been operationalized and assessed.

## 2. Methods

### 2.1. Sources and search strategy

For this research, a specific methodology for systematic reviews was followed: the PRISMA 2020 Statement (Preferred Reporting Items for Systematic reviews and Meta-Analyses), so that a transparent, accurate and complete protocol could be ensured (40). In April 2022, a keyword search was conducted in the Web of Science, PsycInfo and Scopus electronic databases considering the publication period between January 2020 and April 2022. The period considered takes into account the spread of the pandemic outside China, i.e., January 2020, until the time when the articles are searched in the databases, i.e., April 2022. The search terms were (“creativ*” OR “creative thinking” OR “creative performance” OR “creative ability” OR “creative potential”) AND (“undergraduate students” OR “college students” OR “university students” OR “secondary school” OR “high school” OR “higher school”). As in previous reviews of creativity and resilience (41–43), we have considered the terms that best represent the two constructs in scientific literature. For the selection of the age group corresponding to adolescents and young adults, we have referred to the levels of the European Qualifications Framework [EQF; (44, 45)], which corresponds to high school and university students in most countries. Additional parameters included only peer-reviewed, English-language journal articles. The search yielded 676 references in Web of Science, 34 references in PsycInfo and 447 references in Scopus, including 22 duplicates.

### 2.2. Study selection: inclusion and exclusion criteria

To better identify the articles, the PICOS model (46) was used to determine the main characteristics of the studies to be included in the review. The search criteria for inclusion required that, in addition to being peer-reviewed and published in English, the articles (a) reported quantitative data, (b) referred to a population of adolescents and young adults (sample ages 13–25 years), (c) dealt with quantitative assessment of creativity and resilience with (d) outcomes on well-being, quality of life, and posttraumatic growth, and (e) were studies undertaken after the onset of the pandemic (December 2019). Instead, the exclusion criteria were the following: clinical population, Clinical cases, Single case, Clinical trials, Posters, Systematic reviews, Meta-analysis, Conference presentations, Letters to editors, Qualitative studies. The 1,135 articles initially selected from the databases were transferred to the Rayyan program for screening, which was then performed by three independent reviewers. The article screening process that led to the selection is shown in Supplementary Figure S1, schematized through a flow diagram according to the PRISMA 2020 statement guidelines.

The characteristics of the final 4 articles included in the review are shown in Supplementary Table S1.

## 3. Results

The results for each objective are summarized below and explained in more detail in the following section:

- Only one of the four selected studies focused on creativity and resilience in relation to the pandemic period
- The geographical area in which all selected articles were published is Asia, particularly China and Thailand
- The sample of all studies consisted of university students, i.e., mainly young adults
- All articles used resilience as the independent variable and creativity as the dependent variable. All articles included many other variables related to creativity and resilience, such as sense of humor, positive mood, self-esteem and social skills.

## 4. Discussion

### 4.1. How many of the studies were actually conducted during the pandemic period?

Regarding the first objective of the study, only one of the four selected articles addressed creativity and resilience during the pandemic period. This was the study by Zeng et al. (47), which was conducted in China between April and June 2020. Compared to the other three studies, this one has the exact date of data collection, which shows that the research was actually conducted during the pandemic. The lack of an exact date for the administration of the other articles, as well as the complete absence of any reference to the pandemic, lead us to believe that these may be studies conducted prior to the Covid outbreak, especially given the fact that they are studies conducted in Asia, a geographical area particularly affected by the virus. However, it is surprising that there are no other articles in the 2020–2022 biennium with the above inclusion criteria that measured the two variables in relation to the impact of the pandemic and the impact on the well-being and health of adolescents and young adults. Much of the literature that has examined the relationship between creativity and resilience in relation to the post-pandemic period has instead been devoted to socio-economic aspects with a target group of working adults (48–50). An obligatory consideration concerns research during the pandemic and the timing of publication of scientific papers. Indeed, during about half of the 2020s, social isolation and smart working made it difficult to initiate new studies and related data collection unless in an online format (51). This closure meant a drastic reduction in the number of publications during this two-year period, as well as a shift on article topics; mostly focused on certain aspects of public health such as vaccine development, drugs/therapy and the emergence of a new workplace and work culture yet articles on psychological issues mostly began to be published almost a year after the onset of the pandemic, reason why it remains an under-researched area within the Covid context (52).

### 4.2. Geographical area of publication: who was interested in creativity and resilience in the post-pandemic period?

Considering the second objective of the review, it appears that all the articles selected for this review are from Asia, particularly three from the northern and southern provinces of China and one from the city of Bangkok, Thailand. These data are partially consistent with the findings of Hernández-Torrano and Ibrayeva’s (53) review, which covered studies on creativity in education from 1975 to 2019. Their results, while specific to education, show that the three countries with the highest number of publications in these years are the United States, the United Kingdom and China. China thus has proven to be particularly active in research that deals with students and creativity. This is also confirmed by the number of publications on this topic during the last decade, which demonstrates the commitment of Asian countries in the concrete evaluation of policies and strategies to promote creativity in education (54, 55) at the industrial and economic levels (56, 57). On the other hand, as far as the geographical distribution of resilience studies is concerned, to the authors’ knowledge there is no data as precise as in the case of creativity. In Asian countries, however, the focus is mainly on the resilience aspects of the socioeconomic system and less on psychosocial well-being (58).

### 4.3. The reference sample of the studies: adolescents or young adults?

Regarding the third objective of the review, the studies by Zeng et al. (47) and Fan et al. (59) were conducted with a sample of university students, but their age range is not specified. The study by Prasittichok and Klaykaew (60), on the other hand, was conducted with university students aged 18–25, i.e., mostly late adolescents and young adults. According to the information in the articles, only the study by Li et al. (61) was conducted with a sample consisting of adolescents and young adults, i.e., 16–21 years old, but who already belong to the university student group according to the Chinese education system. However, the studies by Fan et al. (59) and Zeng et al. (47) do not refer to the age group, but only to the membership of the sample in the university population, which we can therefore assume to contain a number of adolescents, as in the case of the study by Li et al. (61). In general, it is possible to reflect more on the nature of the sampling than on the actual age group of the study participants. In fact, all of the articles examine creativity and resilience in a sample of university students, a notoriously convenient sample that is readily available for scientific research. University students represent a convenience sample for studies in the humanities, especially for research on well-being and education, because although they are students, they are all of legal age and are also easy to reach *via* online surveys. However, they are often an unrepresentative sample with large age differences and individual differences (62, 63). Internationally (64–66), almost all education research during the pandemic was targeted at university students because only they were accessible for surveys, as opposed to younger students (67).

### 4.4. Operationalization and evaluation of the constructs of creativity and resilience as well as other variables

In relation to the last objective of the review, all studies used self-reported instruments, and three of the four articles used mediating models where the direction of the relationship between creativity and resilience was the same: independent variable resilience and dependent variable creativity. In the study by Li et al. (61), resilience was assessed using the Resilience Scale for Chinese Adolescents (68), while creativity was measured using the Social Creative Questionnaire for University Students [SCQ; (69)]. Other variables that mediated the relationship between resilience and social creativity were sense of humor and positive mood. In the study by Zeng et al. (47), resilience was measured by the construct of post-traumatic growth, using the Posttraumatic Growth Scale (70), while creativity was measured by the Runco Ideational Behaviour Scale (71). Self-efficacy was found to mediate the relationship between post-traumatic growth and creativity, while rumination took on the role of a moderator between self-efficacy and creativity. In the study by Fan et al. (59), the constructs were considered in their social and especially in their team dimensions: thus, resilience was measured with Mallak (72) Team Resilience Scale and creativity with Rego et al. (73) Team Creativity Scale. Other variables considered to mediate the relationship between resilience and team creativity were team creative self-efficacy and team trust. Prasittichok and Klaykaew (60) study, unlike previous studies, pursued a descriptive goal regarding the desired and current states of meta-skills possessed by students. To this end, resilience and creativity, specifically problem solving, were measured using a needs assessment scale based on Kaufman et al. (74) concept of meta-skills and one by Razzetti (75).

## 5. Conclusion

After 2 years into the pandemic, it is not yet possible to predict how isolation, social alienation and distance/hybrid education will affect young people’s education, mental well-being and mental health. However, significant consequences are expected, especially for adolescents and young adults who are particularly vulnerable during crisis situations (76). In the last 2 years, academic literature and the media have paid particular attention to resources of well-being such as creativity and resilience (5, 77), but there is little research on this topic. This review, which screened more than 1,000 articles from 2020 to 2022, found only 4 studies on creativity and resilience in relation to adolescent and young adult well-being (61). Of these studies, only one article actually linked creativity and resilience to Covid-19 outcomes. It might be interesting to analyze whether the publication trend is the same for adults and what other resources with well-being have been studied in the last 2 years in relation to the consequences of a pandemic. All 4 articles were published by Asian research teams, particularly from regions in China and the city of Bangkok. Therefore, future studies could look internationally at which countries have published the most studies on creativity and resilience in the decade before the pandemic to learn more about pre-pandemic trends. In addition, it may be interesting to explore further studies that examine resilience in relation to psychosocial well-being outcomes over the same publication period, as suggested by other meta-analyses on this topic (47, 59, 60, 78). The entirety of the articles presents a sample of university students, but they look at an extended population of about 16–25 years old, i.e., adolescents and young adults. For these reasons, it would be interesting to examine well-being through resilience and creativity in adolescents more systematically during this post-pandemic period. Three of the studies use mediation models to analyze the relationship between resilience (VI) and creativity (VD), while only one describes the level of the two variables as perceived and desired. Although all 4 articles use self-assessment instruments, three of them consider resilience and creativity as individual variables and one as group/team variables. The other variables associated with creativity and resilience in these studies are: positive emotions, sense of humor, self-efficacy, rumination and self-awareness. Future studies could examine which tools are most commonly used to measure creativity and resilience, especially in the last decade, when mobile tools and artificial intelligence have greatly evolved in assessment. This mini-review gives us an opportunity to reflect on the lack of studies that have addressed the issue of youth resources in the form of creativity and resilience since the beginning of the pandemic. The results show us a still underdeveloped interest contrary to what we are told in the media. Much remains to be said about the relationship between creativity and resilience and their contribution to young people’s wellbeing.

## Author contributions

ADL and ER interpreted the data from the literature and wrote the original draft. ADL, LL, and ER reviewed, edited and drafted the manuscript. All authors contributed to the article and approved the submitted version.

## Conflict of interest

The authors declare that the research was conducted in the absence of any commercial or financial relationships that could be construed as a potential conflict of interest.

## Publisher’s note

All claims expressed in this article are solely those of the authors and do not necessarily represent those of their affiliated organizations, or those of the publisher, the editors and the reviewers. Any product that may be evaluated in this article, or claim that may be made by its manufacturer, is not guaranteed or endorsed by the publisher.

## References

[1] Balanzá-Martínez V Atienza-Carbonell B Kapczinski F De Boni RB. Lifestyle behaviours during the COVID-19–time to connect. Acta Psychiatr Scand. (2020) 141:399–400. doi: 10.1111/acps.13177, PMID: 32324252 PMC7264786

[2] Imran N Zeshan M Pervaiz Z. Mental health considerations for children & adolescents in COVID-19 pandemic. Pak J Med Sci. (2020) 36:S67–72. doi: 10.12669/pjms.36.COVID19-S4.2759, PMID: 32582317 PMC7306970

[3] Odriozola-González P Planchuelo-Gómez Á Irurtia MJ de Luis-García R. Psychological effects of the COVID-19 outbreak and lockdown among students and workers of a Spanish university. Psychiatry Res. (2020) 290:113108. doi: 10.1016/j.psychres.2020.113108 32450409 PMC7236679

[4] Rider EA Ansari E Varrin PH Sparrow J. Mental health and wellbeing of children and adolescents during the covid-19 pandemic. BMJ. (2021) 374:n1730. doi: 10.1136/bmj.n1730 34429302

[5] Kapoor H Kaufman JC. Meaning-making through creativity during COVID-19. Front Psychol. (2020) 11:595990. doi: 10.3389/fpsyg.2020.595990, PMID: 33391115 PMC7775492

[6] Elmer T Mepham K Stadtfeld C. Students under lockdown: comparisons of students’ social networks and mental health before and during the COVID-19 crisis in Switzerland. PLoS One. (2020) 15:e0236337. doi: 10.1371/journal.pone.0236337, PMID: 32702065 PMC7377438

[7] Orkibi H Ben-Eliyahu A Reiter-Palmon R Testoni I Biancalani G Murugavel V. Creative adaptability and emotional well-being during the COVID-19 pandemic: an international study. Psychol Aesthet Creat Arts. (2021). doi: 10.1037/aca0000445

[8] Tang M Hofreiter S Reiter-Palmon R Bai X Murugavel V. Creativity as a means to well-being in times of COVID-19 pandemic: results of a cross-cultural study. Front Psychol. (2021) 12:265. doi: 10.3389/fpsyg.2021.601389, PMID: 33767644 PMC7985536

[9] Runco MA Jaeger GJ. The standard definition of creativity. Creat Res J. (2012) 24:92–6. doi: 10.1080/10400419.2012.650092

[10] Cohen LM. Adaptation and creativity in cultural context. Rev Psicol. (2012) 30:3–18. doi: 10.18800/psico.201201.001

[11] Colombo B Valenti C Pizzingrilli P. Conoscere e Usare la Creatività. Milano: EDUCatt-Ente per il Diritto allo Studio Universitario dell'Università Cattolica (2014).

[12] Forgeard MJ. Perceiving benefits after adversity: the relationship between self-reported posttraumatic growth and creativity. Psychol Aesthet Creat Arts. (2013) 7:245–64. doi: 10.1037/a0031223

[13] Metzl ES Morrell MA. The role of creativity in models of resilience: theoretical exploration and practical applications. J Creat Ment Health. (2008) 3:303–18. doi: 10.1080/15401380802385228

[14] Tedeschi RG Calhoun LG. The posttraumatic growth inventory: measuring the positive legacy of trauma. J Trauma Stress. (1996) 9:455–71. doi: 10.1002/jts.2490090305, PMID: 8827649

[15] Alfonso-Benlliure V Moral JCM. Creativity as a" vaccine" for depressed mood: coping and divergent thinking in young adults. Ann Psychol. (2022) 38:209–18. doi: 10.6018/analesps.481761

[16] Conner TS DeYoung CG Silvia PJ. Everyday creative activity as a path to flourishing. J Posit Psychol. (2018) 13:181–9. doi: 10.1080/17439760.2016.1257049

[17] Probst T Chizh A Hu S Jiang L Austin C. Explaining the relationship between job insecurity and creativity: a test of cognitive and affective mediators. Career Dev Int. (2019) 25:247–70. doi: 10.1108/CDI-04-2018-0118

[18] Mohammadi H Asghari Ibrahimabad MJ. The mediating role of creative thinking in the relationship between self-differentiation and self-actualization of couples in Mashhad. Women Fam Cult Educ. (2020) 14:107–24.

[19] Ong AD Bergeman CS Bisconti TL Wallace KA. Psychological resilience, positive emotions, and successful adaptation to stress in later life. J Pers Soc Psychol. (2006) 91:730–49. doi: 10.1037/0022-3514.91.4.730 17014296

[20] Alfonso-Benlliure V Mayordomo T Sales A Mélendez JC. Divergent thinking in older adults: understanding its role in wellbeing. J Happiness Stud. (2021) 22:3285–98. doi: 10.1007/s10902-021-00361-w

[21] Lopez-Persem A Bieth T Guiet S Ovando-Tellez M Volle E. Through thick and thin: changes in creativity during the first lockdown of the Covid-19 pandemic. Front Psychol. (2022) 13:821550. doi: 10.3389/fpsyg.2022.821550, PMID: 35619782 PMC9127054

[22] Karwowski M Zielińska A Jankowska DM Strutyńska E Omelańczuk I Lebuda I. Creative lockdown? A daily diary study of creative activity during pandemics. Front Psychol. (2021) 12:600076. doi: 10.3389/fpsyg.2021.600076, PMID: 33633635 PMC7900138

[23] Mercier M Vinchon F Pichot N Bonetto E Bonnardel N Girandola F. COVID-19: a boon or a bane for creativity? Front Psychol. (2021) 11:601150. doi: 10.3389/fpsyg.2020.601150, PMID: 33536973 PMC7848087

[24] Michinov E Michinov N. Stay at home! When personality profiles influence mental health and creativity during the COVID-19 lockdown. Curr Psychol. (2021) 42:5650–61. doi: 10.1007/s12144-021-01885-3, PMID: 34092985 PMC8163587

[25] Masten AS Wright MO. Resilience over the lifespan: developmental perspectives on resistance, recovery and transformation In: Reich JW Zautra AJ Hall JS, editors. Handbook of Adult Resilience. New York: Guilford (2010). 213–37.

[26] Xu Y Shao J Zeng W Wu X Huang D Zeng Y. Depression and creativity during COVID-19: psychological resilience as a mediator and deliberate rumination as a moderator. Front Psychol. (2021) 12:665961. doi: 10.3389/fpsyg.2021.665961 34025527 PMC8134673

[27] López-Aymes G Acuña SR Ordaz Villegas G. Resilience and creativity in teenagers with high intellectual abilities. A middle school enrichment experience in vulnerable contexts. Sustainability. (2020) 12:7670. doi: 10.3390/su12187670

[28] Thomson E. Resilisence and adaptation In: Runco MA Pritzker SR, editors. Encyclopedia of Creativity, vol. 2. Cambridge, MA, USA: Elsevier (2020). 442–7.

[29] Martínez OL Lozano JN. Rasgos de personalidad y desarrollo de la creatividad. Ann Psychol. (2010) 26:151–8.

[30] Verger NB Urbanowicz A Shankland R McAloney-Kocaman K. Coping in isolation: predictors of individual and household risks and resilience against the COVID-19 pandemic. Soc Sci Humanit Open. (2021) 3:100123. doi: 10.1016/j.ssaho.2021.100123

[31] Aydogdu BN Halil EKSI Celik H. The predictive role of interpersonal sensitivity and emotional self-efficacy on psychological resilience among young adults. Eurasian J Educ Res. (2017) 17:37–54. doi: 10.14689/ejer.2017.69.3

[32] Huang Y Zhao N. Generalized anxiety disorder, depressive symptoms and sleep quality during COVID-19 outbreak in China: a web-based cross-sectional survey. Psychiatry Res. (2020) 288:112954. doi: 10.1016/j.psychres.2020.112954, PMID: 32325383 PMC7152913

[33] Glowacz F Schmits E. Psychological distress during the COVID-19 lockdown: the young adults most at risk. Psychiatry Res. (2020) 293:113486. doi: 10.1016/j.psychres.2020.113486, PMID: 33007682 PMC7518205

[34] Saulle R De Sario M Bena A Capra P Culasso M Davoli M. School closures and mental health, wellbeing and health behaviours among children and adolescents during the second COVID-19 wave: a systematic review of the literature. Epidemiol Prev. (2022) 46:333. doi: 10.19191/ep22.5-6.a542.089, PMID: 36384255

[35] Lattke L De Lorenzo A Rabaglietti E. The relationship between students’ sense of belonging, grit and Anxiety during pandemic times. Revista Romaneasca Pentru Educatie Multidimensionala J. (2022) 14:288–99. doi: 10.18662/rrem/14.4Sup1/673

[36] Pierce M Hope H Ford T Hatch S Hotopf M John A. Mental health before and during the COVID-19 pandemic: a longitudinal probability sample survey of the U.K. population. Lancet Psychiatry. (2020) 7:883–92. doi: 10.1016/S2215-0366(20)30308-4, PMID: 32707037 PMC7373389

[37] Preetz R Filser A Brömmelhaus A Baalmann T Feldhaus M. Longitudinal changes in life satisfaction and mental health in emerging adulthood during the COVID-19 pandemic. Risk Protect Fact Emerg Adulthood. (2021) 9:602–17. doi: 10.1177/21676968211042109

[38] Barbot B Heuser B. Creativity and identity formation in adolescence: a developmental perspective In: The Creative Self. Cambridge, Massachussetts: Academic Press (2017). 87–98.

[39] Barbot B Lubart T. Adolescence, créativité et transformation de Soi. Enfance. (2012) 3:299–312.

[40] Maraolo AE. Una bussola per le revisioni sistematiche: la versione italiana della nuova edizione del PRISMA statement. BMJ. (2021) 372:n7133782057

[41] Aburn G Gott M Hoare K. What is resilience? An integrative review of the empirical literature. J Adv Nursing. (2016) 72:980–1000. doi: 10.1111/jan.12888 26748456

[42] Forgeard MJ Kaufman JC. Who cares about imagination, creativity, and innovation, and why? A review. Psychol Aesthet Creat Arts. (2016) 10:250–69. doi: 10.1037/aca0000042

[43] Said-Metwaly S Van den Noortgate W Kyndt E. Approaches to measuring creativity: a systematic literature review. Creat Theor Res Appl. (2017) 4:238–75. doi: 10.1515/ctra-2017-0013

[44] Brockmann M Clarke L Winch C. Competence and competency in the EQF and in European VET systems. J Eur Ind Train. (2009) 33:787–99. doi: 10.1108/03090590910993634

[45] Méhaut P Winch C. The European qualification framework: skills, competences or knowledge? Eur Educ Res J. (2012) 11:369–81. doi: 10.2304/eerj.2012.11.3.369

[46] Counsell C. Formulating questions and locating primary studies for inclusion in systematic reviews. Ann Intern Med. (1997) 127:380–7. doi: 10.7326/0003-4819-127-5-199709010-00008, PMID: 9273830

[47] Zeng W Zeng Y Xu Y Huang D Shao J Wu J. The influence of post-traumatic growth on college students’ creativity during the COVID-19 pandemic: the mediating role of general self-efficacy and the moderating role of deliberate rumination. Front Psychol. (2021) 12:665973. doi: 10.3389/fpsyg.2021.665973, PMID: 33935927 PMC8079774

[48] Anser MK Yousaf Z Sharif M Yijun W Majid A Yasir M. Investigating employee creativity through employee polychronicity and employee resilience: a glimpse of nurses working in the health-care sector. Eur J Innov Manag. (2020) 25:39–54. doi: 10.1108/EJIM-05-2020-0176

[49] Oparah JC James JE Barnett D Jones LM Melbourne D Peprah S. Creativity, resilience and resistance: black Birthworkers’ responses to the COVID-19 pandemic. Front Sociol. (2021) 6:636029. doi: 10.3389/fsoc.2021.636029, PMID: 33869584 PMC8022614

[50] Sappa V Barabasch A. Theatre technique to foster creative and active problem solving: a resilience-building intervention among in-service teachers. J Adult Contin Educ. (2020) 26:43–60. doi: 10.1177/1477971419842884

[51] Rashid S Yadav SS. Impact of Covid-19 pandemic on higher education and research. Indian J Hum Dev. (2020) 14:340–3. doi: 10.1177/0973703020946700

[52] Haleem A Javaid M Vaishya R Deshmukh SG. Areas of academic research with the impact of COVID-19. Am J Emerg Med. (2020) 38:1524–6. doi: 10.1016/j.ajem.2020.04.022, PMID: 32317202 PMC7158762

[53] Hernández-Torrano D Ibrayeva L. Creativity and education: a bibliometric mapping of the research literature (1975–2019). Think Skills Creat. (2020) 35:100625. doi: 10.1016/j.tsc.2019.100625

[54] Chan S Yuen M. Personal and environmental factors affecting teachers’ creativity-fostering practices in Hong Kong. Think Skills Creat. (2014) 12:69–77. doi: 10.1016/j.tsc.2014.02.003

[55] Tan CS Chin XY Chng STC Lee J Ooi CS. Perceived social support increases creativity: experimental evidence. Int J Environ Res Public Health. (2022) 19:11841. doi: 10.3390/ijerph191811841, PMID: 36142114 PMC9517368

[56] Jyoti J Dev M. The impact of transformational leadership on employee creativity: the role of learning orientation. J Asia Bus Stud. (2015) 9:78–98. doi: 10.1108/JABS-03-2014-0022

[57] Shin SJ Kim TY Lee JY Bian L. Cognitive team diversity and individual team member creativity: a cross-level interaction. Acad Manag J. (2012) 55:197–212. doi: 10.5465/amj.2010.0270

[58] Huang Z. Saxena S. C. Building Forward Better: Enhancing Resilience of Asia and Pacific Economies in a Post-Covid-19 World; (2021). Available at: http://hdl.handle.net/11540/13449 (Accessed November 20, 2022).

[59] Fan M Cai W Jiang L. Can team resilience boost team creativity among undergraduate students? A sequential mediation model of team creative efficacy and team trust. Front Psychol. (2021) 12:2033. doi: 10.3389/fpsyg.2021.604692, PMID: 34177683 PMC8222805

[60] Prasittichok P Klaykaew KK. Meta-skills development needs assessment among undergraduate students. Heliyon. (2022) 8:e08787. doi: 10.1016/j.heliyon.2022.e08787, PMID: 35118206 PMC8792081

[61] Li Y Liu C Yang Y Du Y Xie C Xiang S. The influence of resilience on social creativity: chain mediation effects of sense of humor and positive mood. Psychol Sch. (2022) 59:1609–22. doi: 10.1002/pits.22718

[62] Hanel PH Vione KC. Do student samples provide an accurate estimate of the general public? PLoS One. (2016) 11:e0168354. doi: 10.1371/journal.pone.0168354, PMID: 28002494 PMC5176168

[63] Peterson RA Merunka DR. Convenience samples of college students and research reproducibility. J Bus Res. (2014) 67:1035–41. doi: 10.1016/j.jbusres.2013.08.010

[64] Browning MH Larson LR Sharaievska I Rigolon A McAnirlin O Mullenbach L. Psychological impacts from COVID-19 among university students: Risk factors across seven states in the United States. PloS One. (2021) 16:e0245327. doi: 10.1371/journal.pone.0273938 33411812 PMC7790395

[65] Luo W Zhong BL Chiu HFK. Prevalence of depressive symptoms among Chinese university students amid the COVID-19 pandemic: a systematic review and meta-analysis. Epidemiol Psychiatr Sci. (2021) 30:e31. doi: 10.1017/S2045796021000202, PMID: 33766163 PMC8047400

[66] Tavolacci MP Dechelotte P Ladner J. COVID-19 vaccine acceptance, hesitancy, and resistancy among university students in France. Vaccine. (2021) 9:654. doi: 10.3390/vaccines9060654, PMID: 34203847 PMC8232624

[67] Hanel PH. Conducting high impact research with limited financial resources (while working from home). Meta Psychol. (2020) 4:2560. doi: 10.15626/MP.2020.2560

[68] Hu YQ Gan YQ. Development and psychometric validity of the resilience scale for Chinese adolescents. Acta Psychol Sin. (2008) 40:902–12. doi: 10.3724/SP.J.1041.2008.00902

[69] Hu JY Yang C. A correlative study on social creativity and mental health of college students. Sci Educ Guide. (2010). doi: 10.16400/j.cnki.kjdks.2010.07.024

[70] Geng Y Xu Q Liu H Xu X. Reliability and validity analysis of the Chinese post-traumatic growth scale in multiple trauma survivors. Chin J Nurs. (2011) 46:1003–5. doi: 10.3761/j.issn.0254-1769.2011.10.023

[71] Runco MA Plucker JA Lim W. Development and psychometric integrity of a measure of ideational behavior. Creat Res J. (2000) 13:393–400. doi: 10.1207/S15326934CRJ1334_16

[72] Mallak L. Putting organizational resilience to work. Ind Manag. (1998) 40:8–13.

[73] Rego A Sousa F Pina e Cunha M Correia A Saur-Amaral I. Leader self-reported emotional intelligence and perceived employee creativity: an exploratory study. Creat Innovat Manag. (2007) 16:250–64. doi: 10.1111/j.1467-8691.2007.00435.x

[74] Kaufman RA Rojas AM Mayer H. Needs Assessment: A User's Guide. Englewood Cliffs: Educational Technology (1993).

[75] Razzetti G. The Meta Skills You Need to Thrive in the 21st Century; (2018). Available at: https://liberationist.org/themetaskills-you-need-to-thrive-in-the-21st-century/ (Accessed November 20, 2022).

[76] De Oliveira Araújo FJ de Lima LSA Cidade PIM Nobre CB Neto MLR. Impact of Sars-Cov2 and its reverberation in global higher education and mental health. Psychiatry Res. (2020) 288:112977. doi: 10.1016/j.psychres.2020.112977, PMID: 32302818 PMC7152919

[77] Giovannini E Benczur P Campolongo F Cariboni J Manca AR. Time for Transformative Resilience: The COVID-19 Emergency (No. JRC120489). Luxembourg: Publications Office of the European Union (2020).

[78] Liu JJ Ein N Gervasio J Battaion M Reed M Vickers K. Comprehensive meta-analysis of resilience interventions. Clin Psychol Rev. (2020) 82:101919. doi: 10.1016/j.cpr.2020.101919 33045528

