# Supplementary material for: Creativity and resilience: a mini-review on post-pandemic resources for adolescents and young adults
Source: Front Public Health. 2023 May 24;11:1117539. doi: 10.3389/fpubh.2023.1117539 (PMC10244575; doi:10.3389/fpubh.2023.1117539)
Supplement: Supplementary file 1 [file Presentation_1.pdf]

Supplementary Material

Creativity and resilience: a mini-review on post-pandemic resources for adolescents and young adults

Aurelia De Lorenzo[1*], Lynda S. Lattke[1], Emanuela Rabaglietti[1]

[1]SE-CREA Research Group, Department of Psychology, University of Turin, Turin, Italy

*** Correspondence:** Corresponding Author: aurelia.delorenzo@unito.it

Fig.1 The article screening process Flow Diagram according to the PRISMA 2020 Statement guidelines

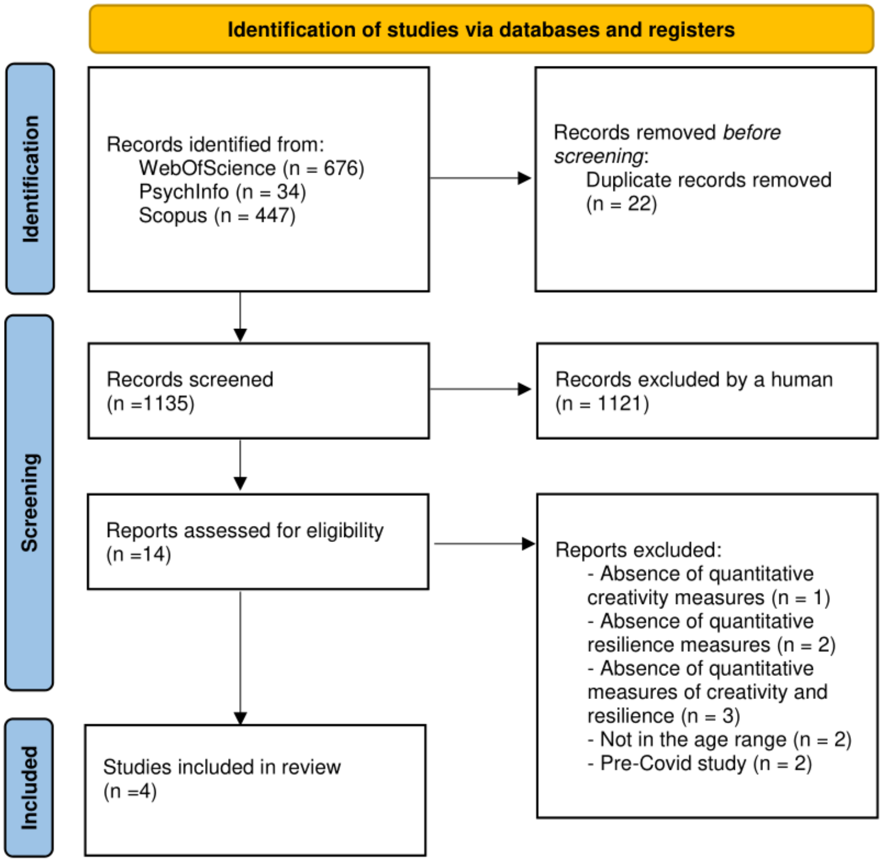

Tab1. Characteristics of the final four articles included in the review

**Authors**	**Titles**	**Years**	**Covid Publication**	**Geaographical area**	**Target sample**	**Models, Instruments and other variables**
Fan, Cai & Jiang	Can team resilience boost team creativity among undergraduate students? A sequential mediation model of team creative efficacy and team trust.	2021	No	Mainland China	University students (no age)	Sequential mediation model;<br>(IV)Resilience: Team Resilience Scale (Mallak,1998)<br>(DV) Creativity: Team Creativity Scale (Rego et al., 2007)<br>Other variables:<br>(Me) team creative self-efficacy; team trust
Li, Liu, Yang, Du, Xie, Xiang, Duan & Hu	The influence of resilience on social creativity: Chain mediation effects of sense of humor and positive mood	2022	No	Northwest China	University students (16-21 y)	Sequential mediation model;<br>(IV)Resilience: Resilience Scale for Chinese Adolescents (Hu & Gan, 2008)<br>(DV) Creativity: Social Creative Questionnaire for University Students (SCQ; Hu & Yang, 2010).<br>Other variables:<br>(Me) sense of humor and positive mood
Prasittichok & Klaykaew	Meta-skills development needs assessment among undergraduate students.	2022	No	Bangkok	University students (18-25 y)	Description of desire and current states of meta skills;<br>Resilience and Creativity: needs assessment Meta-skills scale (Kaufman, Rojas & Mayer,1993; Razzetti, 2018)<br>Other variables: Self-Awareness
Zeng, Zeng, Xu, Huang, Shao, Wu, & Wu	The influence of post-traumatic growth on college students' creativity during the COVID-19 pandemic: the mediating role of general self-efficacy and the moderating role of deliberate rumination	2021	Yes	Guangdong Province, China	University students (no age)	Moderate mediation model;<br>(IV) Resilience: Posttraumatic Growth Scale (Geng et al., 2011),<br>(DV) Creativity: Runco Ideational Behaviour Scale (Runco et al., 2000)<br>Other variables:<br>(Me) self-efficacy<br>(Mo) rumination

Note: IV= independent variable; DV= dependent variable; Me=mediator variable; Mo=moderator variable
